# Bio-interactive nanoarchitectonics with two-dimensional materials and environments

**DOI:** 10.1080/14686996.2022.2054666

**Published:** 2022-03-30

**Authors:** Xuechen Shen, Jingwen Song, Cansu Sevencan, David Tai Leong, Katsuhiko Ariga

**Affiliations:** a Graduate School of Frontier Sciences, The University of Tokyo, Chiba, Japan; b WPI Research Center for Materials Nanoarchitectonics (MANA), National Institute for Materials Science (NIMS), Ibaraki, Japan; c Department of Chemical and Biomolecular Engineering, National University of Singapore, Singapore, Singapore

**Keywords:** Biology, interface, living cell, nanoarchitectonics, two-dimensional material

## Abstract

Like the proposal of nanotechnology by Richard Feynman, the nanoarchitectonics concept was initially proposed by Masakazu Aono. The nanoarchitectonics strategy conceptually fuses nanotechnology with other research fields including organic chemistry, supramolecular chemistry, micro/nanofabrication, materials science, and bio-related sciences, and aims to produce functional materials from nanoscale components. In this review article, bio-interactive nanoarchitectonics and two-dimensional materials and environments are discussed as a selected topic. The account gives general examples of nanoarchitectonics of two-dimensional materials for energy storage, catalysis, and biomedical applications, followed by explanations of bio-related applications with two-dimensional materials such as two-dimensional biomimetic nanosheets, fullerene nanosheets, and two-dimensional assemblies of one-dimensional fullerene nanowhiskers (FNWs). The discussion on bio-interactive nanoarchitectonics in two-dimensional environments further extends to liquid–liquid interfaces such as fluorocarbon–medium interfaces and viscous liquid interfaces as new frontiers of two-dimensional environments for bio-related applications. Controlling differentiation of stem cells at fluidic liquid interfaces is also discussed. Finally, a conclusive section briefly summarizes features of bio-interactive nanoarchitectonics with two-dimensional materials and environments and discusses possible future perspectives.

## Graphical

**Figure:**
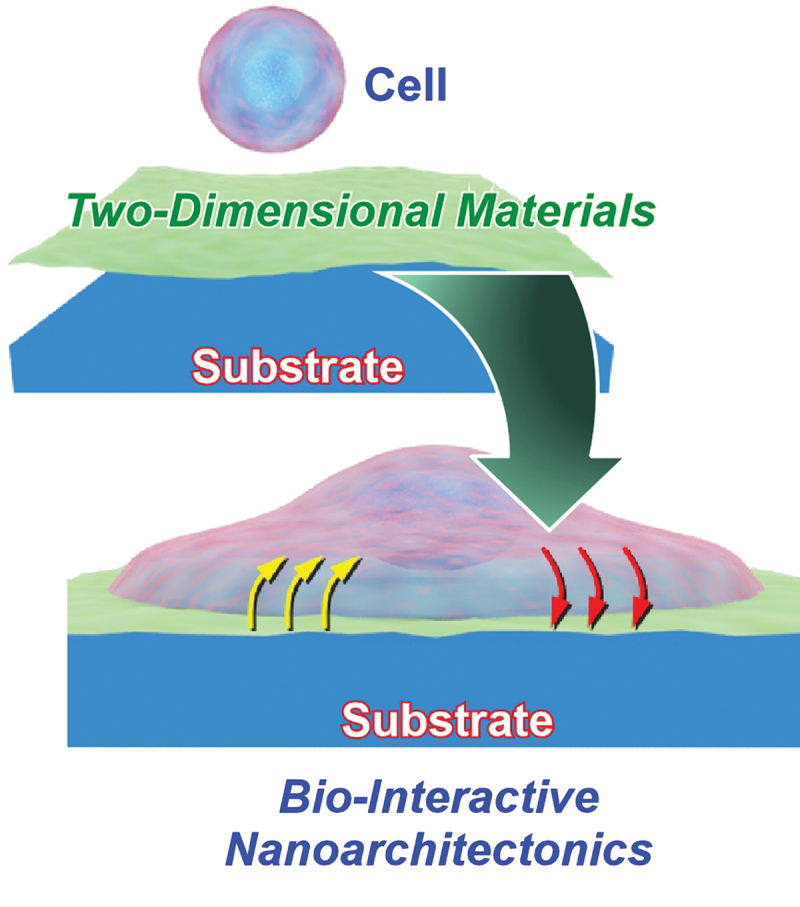

## 1. Introduction: background stories

### 1.1. From nanotechnology to nanoarchitectonics

As one of the remarkable advancements of science and technology in recent decades, nanotechnology and related research fields have attracted much attention. These approaches enable us to precisely observe, evaluate, and manipulate nanoscale objects even at molecular and atomic levels. Novel findings and discoveries made possible by exploring phenomena specifically observable in nanoscale regimes have been continuously reported [1–3]. These advancements are not limited to observation and evaluation. Materials synthesis and fabrication of functional systems based on nanotechnological investigations are also being developed [4–6].

As Nakamura, Harano, and co-workers recently demonstrated, sophisticated electron microscopy techniques (single-molecule atomic-resolution real-time electron microscopy, SMART-EM) enable us to observe shuttling, rotating, and interacting motions of a single fullerene (C_60_) molecule entrapped within a vibrating carbon nanotube in real-time with sub-angstrom resolution and sub-millisecond precision [7]. The SMART-EM techniques can be used to observe single-molecule-level chemical reactions with the aid of the fishhook technique [8]. Introduction of aromatic groups onto the graphitic surface of carbon nanohorns with positive and negative curvature was detected. Immobilization of molybdenum dioxo catalyst at the graphitic surfaces of carbon nanohorns was directly confirmed by the SMART-EM technique [9]. The nanoarchitected catalyst showed high activities in several reactions including N-oxide reductions, polyethylene terephthalate hydrogenolysis, and reductive carbonyl coupling. Visualization of plasmon-induced reactions with single-molecule-level precision was enabled by a scanning tunnelling microscope (STM) as reported by Kazuma [10]. With aid of density functional theory calculations, bond dissociation reactions caused by plasmon excitation at a nanoscale gap between a metal substrate and the STM tip with light irradiation can be analysed. Narita, Müllen, and co-workers are actively working on on-surface synthesis of large polycyclic aromatic hydrocarbon and graphene nanoribbons from well-defined molecular sources [11]. This series of approaches are supported by high-resolution observation through noncontact atomic force microscopy [12]. As recently demonstrated by Kawai and co-workers, coupling reactions directly mediated by small tip motion is possible; additional reactions with bromine atoms and fullerene molecules to specific surface positions using a scanning probe tip were realized with single-molecule-level precision [13]. Device operation with molecular and atom-level phenomena have been demonstrated as atom switches [14,15] and molecular memory [16,17], respectively, by Aono and co-workers. In a recent review article by Yamashita, confinement of single-molecule magnets into designed nanospaces formed by single-walled carbon nanotube and metal-organic framework (MOF) is suggested for application in advanced devices such as high-density memory devices and quantum computing [18].

On the other hand, methodologies based on well-established sciences such as organic chemistry [19–21], supramolecular chemistry [22–24], coordination chemistry [22–27], bio-related sciences [28–30], and other materials sciences [31–33] have continuously contributed to the production of functional material systems. Undoubtedly, syntheses and fabrications of functional materials are indispensable keys to satisfy various social demands in energy [34–36], environmental [37–39], and biomedical targets [40–42]. As seen in solar cells [43–45], fuel cells [46–48], batteries [49–51], photocatalytic conversions [52–54], and supercapacitors [55–57] for energy demands, sensing/detection [58–60], removal [61–63], and degradation [64–66] of problematic materials for environmental issues, drug delivery [67–69] and various therapies [70–72] in biomedical applications, control over precise structures and organizations is as important as intrinsic properties of materials themselves. Therefore, the introduction of nanotechnological fundamentals to materials science is crucial for further developments of materials science to meet recent social demands. A new paradigm and concept to couple nanotechnology and other research fields in materials science with nanostructure regulation needs to be established as a post-nanotechnology concept [73]. This task is assigned to an emerging concept: nanoarchitectonics [74,75].

**Figure 1:**
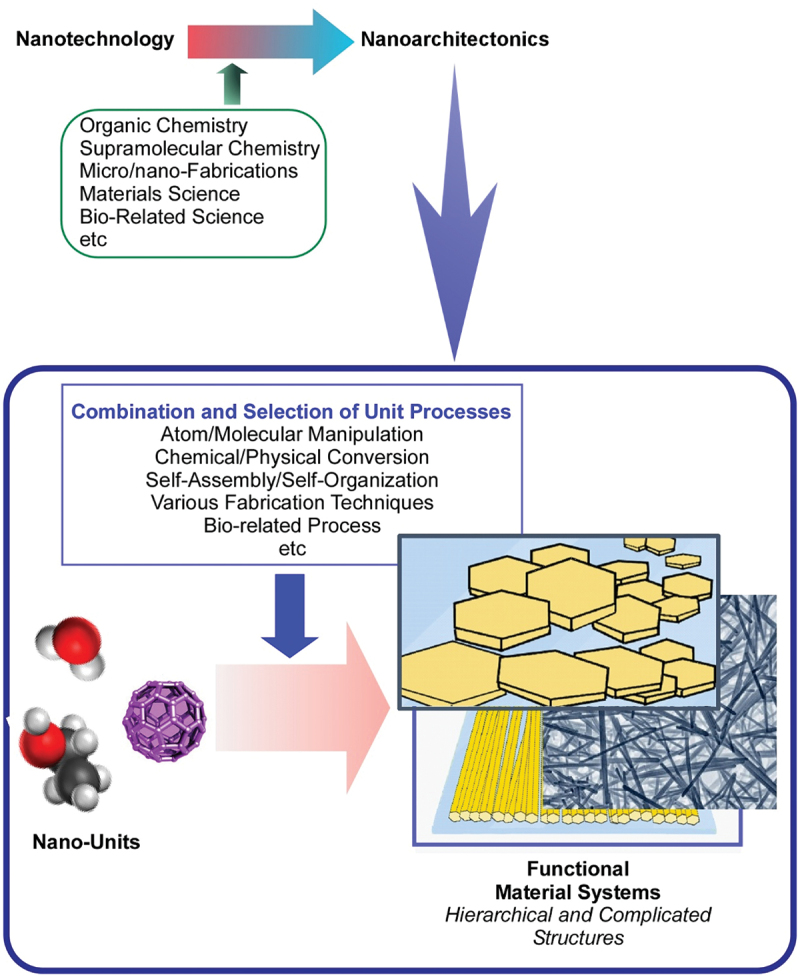
Outline of nanoarchitectonics strategy.

Like the proposal of nanotechnology by Richard Feynman [76,77], the nanoarchitectonics concept was initially proposed by Masakazu Aono [78,79]. The nanoarchitectonics strategy conceptually fuses nanotechnology with other research fields including organic chemistry, supramolecular chemistry, micro/nano-fabrication, materials science, and bio-related sciences (Figure 1) [80,81]. This methodology aims to produce functional materials from nanoscale components by combining and selecting individual processes such as atom/molecule manipulation, chemical/physical conversion, self-assembly/self-organization, various fabrication techniques, and bio-related processes [82]. Because the nanoarchitectonics concept is very general, related approaches can be applied to a wide range of materials. Even in recent publications, it can be found that the nanoarchitectonics concept is utilized for rather traditional materials targets such as organic modification of oxide-based layered materials [83] and composite materials for removal of cesium ions from water [84] as well as forefront areas like coordination asymmetry [85] and DNA/RNA-based molecular machines [86]. In an analogy to the theory of everything in physics, nanoarchitectonics can be regarded as the strategy of everything in materials science [87]. Applications of the nanoarchitectonics approach have been proposed in a wide variety of functional targets including functional material production [88,89], structural regulations [90,91], catalysts [92,93], sensors [94,95], devices [96,97], energy-related applications [98,99], solutions for environmental problems [100,101], basic bioscience [102,103], and biomedical applications [104,105].

However, nanoarchitectonics strategies may include features different from conventional materials fabrications. Unlike macroscopic and microscopic phenomena, nanoscale interactions between objects (atoms, molecules, and materials) are exposed to various factors of uncertainty such as thermal fluctuations, statistic distributions, complicated mutual interactions, and quantum effects. Summed effects in materials are not simply additive but can also be synergistic. Materials nanoarchitectonics may need to consider effects of such uncertainties in their constructions and functions [106]. This situation is reminiscent of biological systems, in which various functional elements work together under thermal fluctuations [107,108]. Another distinct feature of nanoarchitectonics processes would be its suitability for forming hierarchical structures [109]. Unlike self-assembly processes at equilibrium, nanoarchitectonics can combine various construction processes. Formation of hierarchical structures with asymmetrical and/or anisotropic organization becomes possible in nanoarchitectonics-based materials constructions. In most biological functional systems, hierarchical organizations are fundamental for creating rational flows of energy, electrons, and information. Therefore, nanoarchitectonics approaches have the potential to form bio-like highly functional materials systems.

### 1.2. Two-dimensional materials and environments

Among various examples in materials nanoarchitectonics, approaches specific to two-dimensional materials and two-dimensional environments (interfacial environments) exhibit exciting possibilities [110–112]. One of the motivations for active research on two-dimensional nanoarchitectonics is rapid advancements in two-dimensional materials. Various two-dimensional materials including graphene families [113,114], two-dimensional semiconductors [115,116], various nanosheets [117,118], two-dimensional organic polymers [119,120], two-dimensional coordination polymers [121,122], and organic thin films [123–125] have been widely researched. They are promising components for materials nanoarchitectonics. In recent reports, various functions are demonstrated by materials constructed from two-dimensional materials as structural components. For example, Tajima et al. reported long lifetimes of charge-separate states in mixed-dimensional (zero-dimensional/two-dimensional) van der Waals heterojunctions of anthracene physically adsorbed on few-layer MoS_2_ nanosheets [126]. The observed properties would be useful for efficient photocatalysts, photovoltaics, and optoelectronics applications. Vinu and co-workers successfully synthesized hybrids of two-dimensional mesoporous fullerene and carbon materials for energy-related applications such as Li-ion batteries and supercapacitors [127]. For environment-oriented applications, photodegradation of picric acid by two-dimensional materials made from germananes terminated with hydrogen and methyl groups was demonstrated by Sturala, Sofer, and co-workers [128]. Surface plasmon resonance sensors were nanoarchitected from two-dimensional MoS_2_ nanosheets modified with the supramolecule calix[4]arene as demonstrated by Hu, Chen, and co-workers [129]. This sensor system was utilized for detection of bovine serum albumin antibodies. As summarized in a recent review article by Choi and co-workers, two-dimensional materials can be used in various imaging technologies including magnetic resonance imaging, computed tomography and positron emission tomography as well as conventional optical imaging [130]. These imaging methods are expected to be utilized in image-guided and precision therapy. Free-standing conductive two-dimensional polymer nanosheets were recently reported by Fujie and co-workers [131]. The nanoarchitected polymer nanosheets can make good contact with unevenly structured surfaces, such as the veins of plant leaves. Integration of a Bluetooth system into the polymer nanosheets enables wireless biopotential measurement in plants. As Li and co-workers summarized in a recent review article, stimuli-responsive drug delivery systems can be nanoarchitected from various two-dimensional nanosheets [132]. As Oaki and Igarashi recently demonstrated, application of materials informatics for predicting exfoliation processes to prepare two-dimensional materials has also been proposed [133].

Interfacial environments as two-dimensional working media also promote understanding of various materials phenomena and nanoarchitectonics-based materials synthesis [134,135]. Particularly, dynamic interfaces such as an air–water interface provide unusual opportunities for molecular controls including molecular machine handling by hand-motion-like macroscopic mechanical input [136–138] and mechanical tuning of molecular receptor performances [139,140]. These unique functions are created by extremely different motional freedoms between lateral and transverse directions of the fluidic interfacial media. In addition, the heterogeneous dielectric nature of the air–water interface induces drastic enhancements in molecular recognition capability of aqueous guest molecules [141,142]. Although hydrogen-bond-based molecular recognition is highly suppressed in bulk water, an incredible increase in binding efficiency (10^6^ − 10^7^ times in some cases) is observed at the interfacial media [143]. This leads to efficient sensing system designs and a true understanding of biological phenomena [144]. Although molecular recognition in biological systems occur in high dielectric aqueous media, aqueous media are not ideal media for hydrogen bonding for external guests. This seemingly contradictory situation can be resolved by considering interfacial environments. Molecular recognition in biological systems mostly occur at interfaces with aqueous medium as is the case for the surface of cell membranes, inner surfaces of proteins, and macromolecular interfaces of nucleic acids. Two-dimensional interfacial media can be regarded as powerful biomimetic environments for bio-inspired nanoarchitectonics [145,146].

Accordingly, there has been a lot of interest in analysing and understanding various phenomena at interfacial environments as seen in recent literature. Investigations on two-dimensional lipid membranes formed at interfacial environments have been progressing continuously. For example, Ishibashi and co-workers elucidated diffusion coefficients of trans-stilbene embedded within a lipid bilayer membrane of dimyristoylphosphatidylcholine formed at a silica–water interface [147]. Two different observed values of effective viscosity imply local inhomogeneity of the lipid membrane as a fluidic two-dimensional medium. Oxidation of unsaturated phospholipids with various head groups under low-level ozone environments at the air–water interface was systematically investigated using heterodyne-detected sum frequency generation spectroscopy as reported by Inoue, Ye, and co-workers [148]. Makiura et al. investigated two-dimensional assemblies of amphiphilic mesogens with different peptide chains at the air–water interface [149]. The knowledge obtained from these model studies is useful for understanding the ordering of liquid crystalline materials at various interfaces. Kinjo et al. reported theoretical approaches for molecular dynamics simulation of fractures at interfaces between polyphenylene sulfide and aluminum oxide under tensile force [150]. It was revealed that fracturing between the polymer and solid is initiated by small voids forming within the polymer layer.

Two-dimensional interfacial media have been used for materials nanoarchitectonics to synthesize new types of two-dimensional materials and related functional materials systems. In fact, nanoarchitectonics synthesis and organization of ultrathin two-dimensional materials have been demonstrated in one-dimensional supramolecular polymerization of two-dimensional DNA origami nanosheets [151], integrated fabrication of optically regulated one-dimensional supramolecular fibers [152], carbon nanosheet synthesis from carbon nanoring molecular precursors by vortex LB method [153], and highly oriented two-dimensional nanofilms of polymeric semiconductors by super-high-temperature LB method [154,155]. As summarized in a recent review article by Wan and co-workers, various two-dimensional interfacial media such as gas–liquid, liquid–liquid, and liquid–solid interfaces are useful for synthesizing two-dimensional covalent organic frameworks (COFs) without limitations associated with their poor solubility [156]. Particularly, interfaces formed with insoluble solvents (liquid–liquid interfaces) provide opportunities to form small shape-defined crystals arising from drastic changes in material solubility. For example, the dynamic liquid–liquid interfacial precipitation method results in macaroni-shaped fullerene (C_60_) crystals that can be converted into mesoporous carbon tubes upon high-temperature heat treatment at 900°C [157]. The formed carbon materials exhibited nice supercapacitor performances with excellent stability even after 10,000 charging–discharging cycles. Niidome, Kurawaki, and co-workers demonstrated the formation of gold-silver bimetallic thiolate complexes at the interface between octanethiol and water accompanied with luminescence conversion from red to blue [158].

These examples demonstrate the strikingly important roles of two-dimensional materials and environments in materials nanoarchitectonics. Anisotropic structures and dielectrically heterogeneous natures of such two-dimensional matter are surely beneficial for various applications including popular energy-related applications and bio-related investigations.

### 1.3. Objectives of this review article

These background descriptions suggested several important points for producing functional systems in current science and technology of advanced materials. Following sufficient increase in control over nanostructure in functional materials, a novel methodology, nanoarchitectonics, has emerged as the post-nanotechnology concept. In the nanoarchitectonics approach, functional material systems are architected from nanoscale unit-components using various processes including self-assembly/self-organization. Nanoarchitectonics processes have features reminiscent of biological systems, where synergistic material interactions under nanoscale fluctuations play important roles in organizing hierarchical structures for advanced functions. It may even be said that real biological systems are products of natural nanoarchitectonics processes. Therefore, creation of bio-like highly functional material systems would be an ultimate goal of nanoarchitectonics approaches.

From the viewpoint of producing new artificial materials systems with biological-level high functionality, bio-interactive nanoarchitectonics must be considered. Bio-related functional material systems are usually based on bioactive interactions as seen in recent examples such as ultrasound-activated therapeutics [159], molecular hybridization for detection and imaging of biomarkers inside brains afflicted with Alzheimer’s disease [160], interactive materials systems for detecting microcystin-leucine arginine as one of the most toxic and harmful freshwater toxins [161], nanostructured materials systems with localized surface plasmon resonance for sensing cytokine [162], and structurally designed peptide nucleic acids for interactive detection of specific forms and sites of DNA and RNA [163].

**Figure 2:**
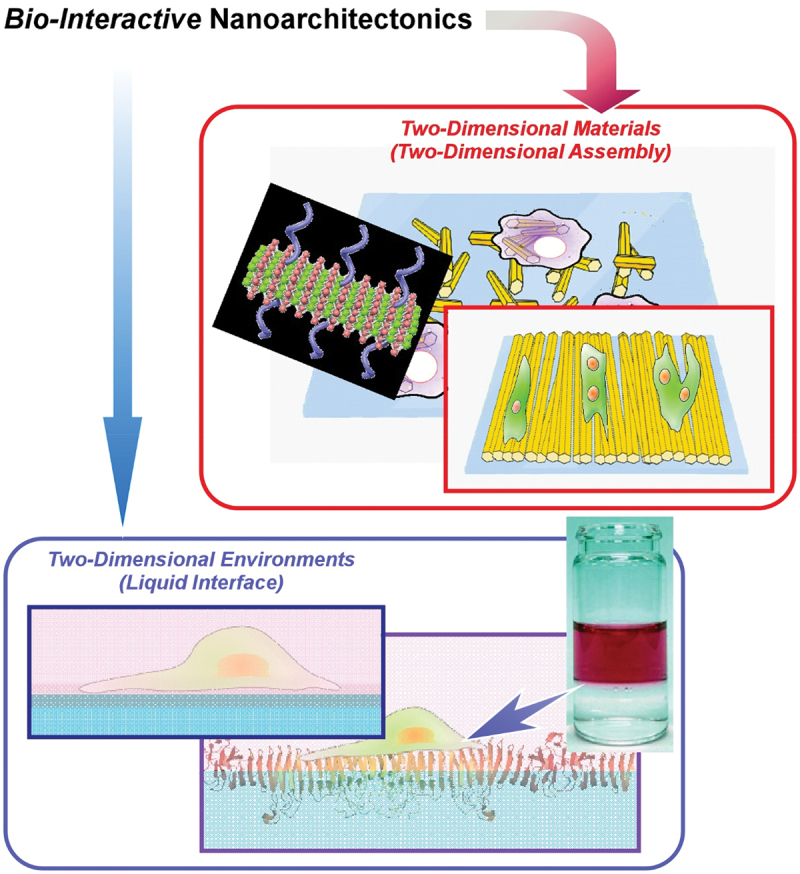
Objectives of this review article: bio-interactive nanoarchitectonics with two-dimensional materials and/or at two-dimensional environments.

Although various nanoscale units are architected in the bio-interactive functional materials in the previous examples, this review focuses especially on two-dimensional materials and environments (Figure 2). As described in the previous section, two-dimensional materials are star-players in recent nanomaterials sciences, and two-dimensional (interfacial) environments are advantageous media for molecular interactions including biological interactions. In this review article, several examples are selected to explain bio-interactive nanoarchitectonics coupled with two-dimensional materials and environments. Following general examples of nanoarchitectonics of two-dimensional materials for energy storage, catalysis, and biomedical applications (section 2), bio-related applications with emerging two-dimensional materials such as two-dimensional biomimetic nanosheets, fullerene nanosheets, and two-dimensional assemblies of one-dimensional fullerene nanowhiskers (FNWs) are explained (section 3). Further discussions on bio-interactive nanoarchitectonics in two-dimensional environments extend to liquid–liquid interfaces such as fluorocarbon–medium interfaces and viscous liquid interfaces as new frontiers of two-dimensional environments for bio-related applications (section 4). In the latter section, controlling differentiation in stem cells at fluidic liquid interfaces is discussed. Finally, a conclusive section briefly summarizes features of bio-interactive nanoarchitectonics with two-dimensional materials and environments and discusses possible future perspectives.

## 2. General applications of nanoarchitectonics of two-dimensional materials

Following the discovery of graphene, many types of two-dimensional nanomaterials with monolayer thickness have been developed and explored because of their unique properties endowed by the monolayer conformation [164,165]. With the excitement of discovering these novel properties, earlier research mainly focused on the discrete properties of nanosheets. However, as the subject of two-dimensional nanomaterials became further established, the potential for assembling these two-dimensional nanosheets into three-dimensional shapes and hybrid structures gathered interest. Here, we summarise the main areas of application for such assemblies with focus on commonly used two-dimensional nanomaterials.

### 2.1. Energy storage and catalysis

Two-dimensional nanomaterials have shown promise for energy storage due to their good electrical conductivity and large surface area providing abundant active sites for ions to intercalate [166,167]. Nonetheless, it was also discovered that a number of factors limit their efficacy such as their tendency to aggregate and electrochemical degradation of active sites [168,169]. Hetero-structures made up of two-dimensional nanosheets have been developed to eliminate or minimize limiting factors and fabricate enhanced energy storage devices.

**Figure 3:**
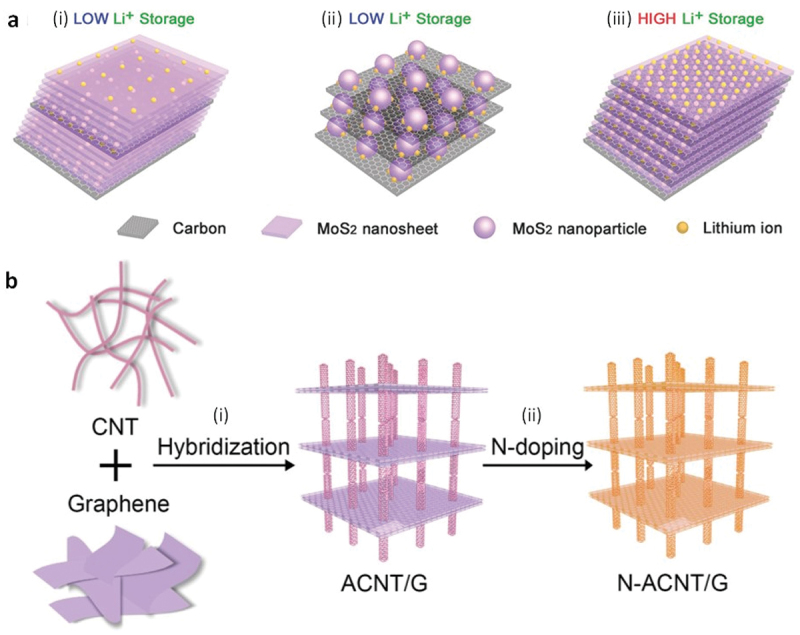
Assemblies of two-dimensional nanomaterials are good anode or cathode candidates for lithium batteries. a) Schematic illustrating the concept of the rational design of MoS_2_/m-C nanosheet superstructure for creating ideal MoS_2_/c atomic interfaces to enhance lithium-ion storage. Reproduced with permission [168] 2015, Wiley-VCH. b) Conceptual scheme of the design of N-ACNT/G hybrids with graphene and aligned CNTs as building blocks. (i) Structural hybridization of aligned CNTs and graphene via catalytic growth on bifunctional natural catalysts; (ii) in situ nitrogen doping for moderating chemical modification of the carbon scaffolds. Reproduced with permission [169] 2014, Wiley-vch.

A sandwich-like assembly of two-dimensional monolayer MoS_2_ nanosheets with mesoporous carbon between each layer of nanosheet (MoS_2_/m-C) was engineered for lithium-ion storage (Figure 3) [170]. The MoS_2_/m-C structure was obtained by amidating dopamine onto oleic-acid-protected monolayer MoS_2,_ followed by self-polymerizing dopamine and finally annealing the obtained structure at 850°C for 2 h. Engineered MoS_2_/m-C was shown to solve some of the common problems in MoS_2_-based anode electrode materials for lithium-ion batteries such as low conductivity of MoS_2_ in the *c*-direction and aggregation and restacking of MoS_2_ nanosheets. This hybrid structure also provided the largest interfacial contact for Li ion storage among MoS_2_-based anode electrode materials. Another sandwich-like structure consisting of carbon nanotubes (CNTs) and graphene nanosheets was engineered for lithium-sulfur (Li-S) batteries (Figure 3) [169]. Vertically aligned CNTs were anchored onto the graphene nanosheets (ACNT/G), creating a hierarchical structure. The obtained architecture provided effective ion diffusion channels and three-dimensional electron transfer pathways. Nitrogen doping (N-ACNT/G) enhanced the electrochemical performance by introducing additional active and defect sites. Engineered N-ACNT/G demonstrated improved cyclic and rate performance when used as the cathode in Li-S batteries.

**Figure 4:**
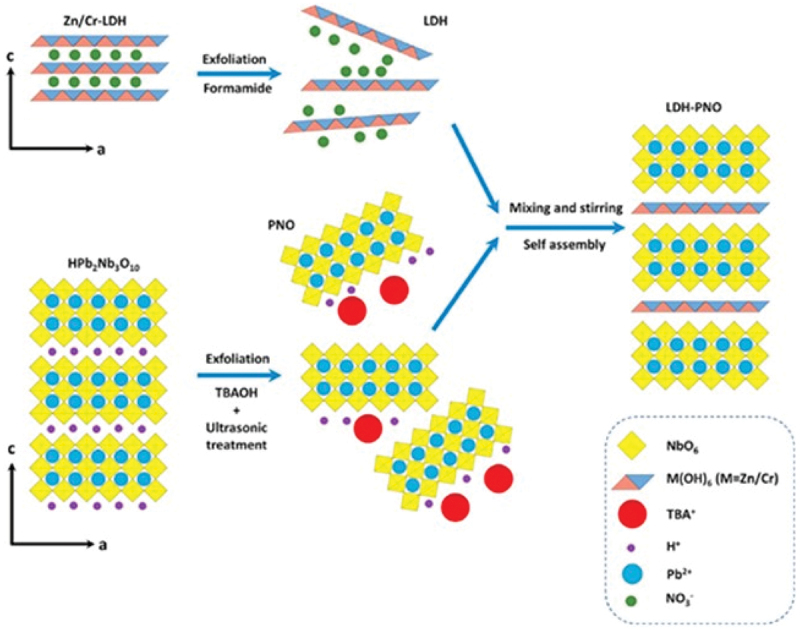
Schematic illustrating the synthesis of LDH-PNO for the photocatalytic oxygen generation. Reproduced with permission [172] 2021, Elsevier. Hybrid structure was self-assembled by layer-by-layer stacking of Zn/Cr-layered double hydroxide (Zn/cr-LDH) and lead niobate (HPb_2_Nb_3_O_10_) nanosheets.

As is the case for energy storage applications, the true potential of two-dimensional materials as catalysts is hindered by a number of factors. Using strategic architectures of hybrid structures, better catalysts can be obtained from two-dimensional nanomaterials [170,171]. Zn/Cr-layered double hydroxide (Zn/Cr-LDH) and lead niobate (HPb_2_Nb_3_O_10_) nanosheets were self-assembled into layer-by-layer stacked formation (LDH-PHO) for photocatalytic oxygen generation (Figure 4) [172]. In a spontaneous electrostatic process, negatively charged HPb_2_Nb_3_O_10_ nanosheets self-assembled alternately with positively charged Zn/Cr-LDH nanosheets. The assembled hetero-structure demonstrated enhanced photocatalytic activity compared to lone Zn/Cr-LDH nanosheets due to suitable band alignment and large contact area between the layered nanosheets.

### 2.2. Biomedical applications

Two-dimensional nanomaterials have greatly contributed to the development of diagnostic and therapeutic tools such as biosensors, drug delivery systems and photothermal and photodynamic therapy agents [173,174]. Exploration into the assemblies and hybrid structures of these two-dimensional nanomaterials has widened the potential biomedical applications of these materials.

**Figure 5:**
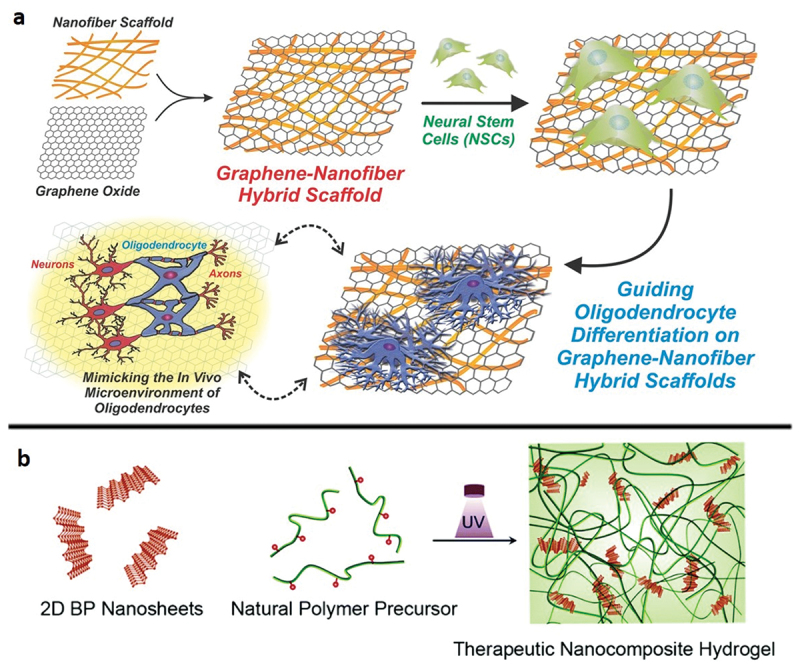
Scaffolds for tissue engineering applications can be constructed by the assembly of two-dimensional nanomaterials. a) Schematic diagram depicting the fabrication and application of graphene-nanofiber hybrid scaffolds. Polymeric nanofibers (composed of polycaprolactone) generated using electrospinning were subsequently coated with GO and seeded with NSCs. NSCs cultured on the graphene-nanofiber hybrid scaffolds show enhanced differentiation into oligodendrocyte lineage cells. Reproduced with permission [176] 2014, Wiley-VCH. b) Schematic illustration of the preparation of the therapeutic hydrogel from a GelMA prepolymer and BP nanosheets. Reproduced with permission [178] 2019, Royal Society of chemistry.

Three-dimensional assemblies and hybrid structures involving two-dimensional nanosheets were mainly explored for the fabrication of tissue engineering scaffolds. Besides good photothermal and conductive properties, two-dimensional nanosheets were impactful especially due to their ability to induce cell differentiation without adding differentiation inducers in the culture media [175,176]. The potential of MoS_2_ and reduced graphene oxide (rGO) nanosheets as electrically conductive agents and nanotopographical stimuli for fabricating cardiac tissue engineering scaffolds was evaluated. The scaffold was constructed by introducing MoS_2_ and rGO nanosheets into silk fibroin nanofibers. Induced pluripotent stem cells (IPSCs) transfected with TBX18 gene were cultured on this scaffold. Through a series of experiments assessing the mechanical properties of the structure and cultured IPSCs, it was determined that the scaffold could be a good candidate for cardiac tissue engineering [175]. In another study, GO nanosheet coated polycaprolactone (PCL) nanofiber scaffold was utilised for neural tissue engineering (Figure 5) [176]. Differentiation of neural stem cells (NSCs) into oligodendrocytes was observed to be dependent on GO concentration. It was shown that the GO coating provided a suitable surface for cell adhesion and promoted neuronal electrophysiology due to its good conductivity.

Two-dimensional black phosphorus (BP) is especially preferred for bone regeneration applications due to its phosphorus (P) content. BP is easily broken down into phosphorus (P), a vital element in bones, upon interacting with oxygen, visible light and water. Hence, different structures were combined with two-dimensional BP for bone tissue engineering. In one such example, a 3D printed bioglass scaffold was modified with two-dimensional BP nanosheets for photothermal therapy of osteosarcoma and ensuing bone regeneration [177]. In another study, the photothermal capability of two-dimensional BP was utilised for its antibacterial effect, while the two-dimensional BP hydrogel nanocomposite promoted mineralization and supported crosslinking networks for osteogenesis and bone tissue formation (Figure 5) [178]. Another use of the photothermal capability of two-dimensional BP in bone tissue engineering applications was demonstrated through the fabrication of an osteoimplant; two-dimensional BP nanosheets were combined with poly(lactic-co-glycolic acid) (PLGA) and implanted into the hind legs of rats. Osteogenesis was observed upon periodic near-infrared (NIR) laser irradiation [179].

**Figure 6:**
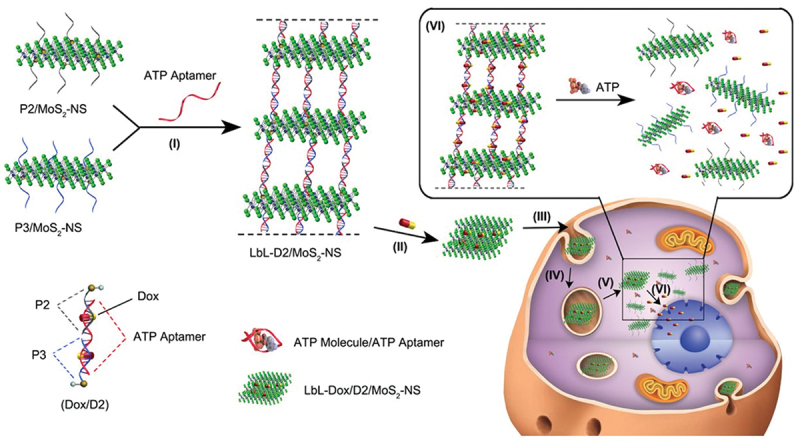
Stimuli-Responsive anti-cancer drug delivery system is formed by layer-by-layer self-assembly of MoS2 nanosheets. Schematic illustration of formation of multilayer Dox/D2/MoS_2_-NS and their intracellular Dox release process. (I) ATP-aptamer-induced LbL assembly; (II) Dox loading in multilayer nanostructures; (III) in vitro treatment of Dox/D2/MoS_2_-NS; (IV) cell uptake through endocytosis; (V) endosomal/lysosomal escape; (VI) ATP-induced Dox release in the cytosol. Reproduced with permission [180] 2017, American Chemical Society.

In addition to tissue engineering applications, other biomedical uses of two-dimensional assemblies, such as drug delivery and gene delivery, have been explored. Li et al. designed a layer-by-layer assembly of MoS_2_ nanosheets mediated by DNA oligonucleotides for the delivery and stimuli-responsive release of the chemotherapeutic drug Doxorubicin (DOX) (Figure 6) [180]. Thiol-terminated single-stranded DNA (ssDNA) was anchored onto the sulphur vacancies on the MoS_2_ nanosheets. Self-assembly and stimuli-responsive capabilities were achieved using DNA-based aptamers as linkers. DOX was loaded into the layered structure through its ability to preferentially bind GC base-pair duplexes. The chosen ATP-responsive aptamer enabled the release of carried DOX at the ATP-rich tumour site by dissociating from the DNA oligonucleotides and preferentially binding to ATP molecules instead. MoS_2_ nanosheets acted as a protective shield for the cargo while allowing small ATP molecules to infiltrate for stimuli-responsive effect. Paul et al. utilised GO incorporated into injectable low-modulus methacrylated gelatin (GelMA) hydrogel for gene delivery to myocardial tissue [181]. The cargo of pro-angiogenic human vascular endothelial growth factor plasmid DNA (pDNA_VEGF_) was bound to the surface of GO prior to its combination with GelMA. *In vitro* and *in vivo* studies showed enhanced cardiac activity in cells transfected by the delivered gene therapy.

## 3. Emerging two-dimensional materials in bio-related applications

### 3.1. Two-dimensional biomimetic nanosheets

As a new subcategory of two-dimensional nanomaterials, biomimetic nanosheets are being developed by layering biologically obtained nanomaterials with inorganic nanomaterials in two-dimensional conformations. In this technique, naturally soft bio-nanomaterials such as cell membrane fragments are stiffened to maintain two-dimensional shape through added support from hard inorganic nanoparticles.

In one earlier example, researchers layered red blood cell (RBC) membranes with MoSe_2_ nanosheets for cancer immunotherapy [182]. RBC membranes increased circulation time of nanoparticles by providing immune evasion. MoSe_2_ provided near-infrared (NIR) laser-induced ablation of the primary tumour by photothermal therapy (PTT), while immunotherapy was achieved through activating the inherent immune system through antigens released from ablated tumour cells.

**Figure 7:**
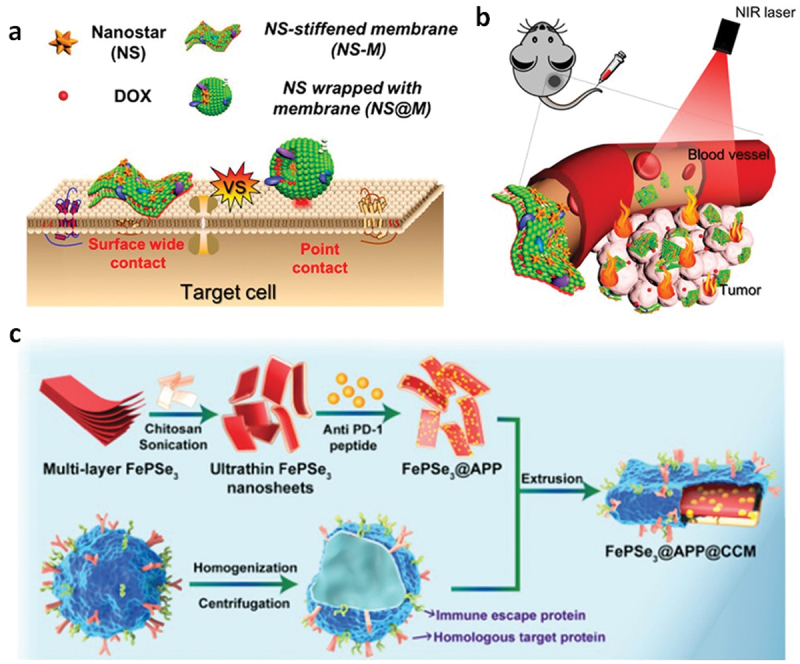
Combining biomimetic two-dimensional nanomaterials with inorganic nanomaterials is an emerging technique for designing new diagnostic and therapeutic nanomaterials. a) Schematic illustration of the stiffened nanomembrane and the three-dimensional nanosphere’s interaction with target cell surface. b) Schematic illustration of the drug-loaded stiffened nanomembrane for tumor ablation and chemotherapy. Reproduced with permission [183] 2020, American Chemical Society. c) Schematic illustration of the design and preparation of FePse_3_@app@ccmnss. Reproduced under terms of the CC-BY license [185].

In another example, the advantage of two-dimensional bio-nanomaterials over their spherical counterparts in anti-tumour applications was demonstrated (Figure 7) [183]. For this purpose, two-dimensional cell membrane nanoparticles (NS-M) were prepared by layering gold nanostars (NS) with cell membrane, while three-dimensional cell membrane nanoparticles (NS@M) were prepared by encapsulating NS into cell membrane vesicles. Through comparing the two modalities, it was shown that NS-M has superior anti-tumour efficacy. Due to its flattened shape, NS-M could more effectively accumulate in the tumour site and attach to tumour cells strongly, which in turn increased its anti-tumour efficacy. Similarly, structured nanosheets were prepared in another work by combining *E. coli* membrane nanosheets with gold nanoparticles (AuMNs) [184]. AuMNs were successful in PTT-induced killing of tumours and worked as immunologic adjuvants to promote anti-PD-L1 efficacy.

In a more recent study, two-dimensional FePSe_3_ metal phosphorous trichalcogenides were combined with CT26 cell membrane and loaded with anti-PD-1 peptide (APP) for imaging and therapy on cancer cells (Figure 7) [185]. FePSe_3_ acted as magnetic resonance imaging (MRI) and photoacoustic imaging (PAI) agents while also contributing to anti-tumour activity by inducing PTT under NIR laser irradiation. The anti-tumour effect was enhanced by T cell activation through the loaded APP. CT26 cell membrane worked as a targeting agent, helping the constructed two-dimensional nanoparticle to homotypically locate cancer cells.

### 3.2. Fullerene nanosheets

C_60_ fullerene is an allotrope of sp^2^ carbons arranged in a spherical network [186,187]. In solution, C_60_ fullerenes self-assemble into crystal structures defined by intermolecular forces. C_60_ fullerene’s size (1 nm diameter) and zero-dimensional structure make it a versatile molecular building block. Using liquid–liquid interfacial precipitation (LLIP) to control structure, various one-dimensional to three-dimensional assemblies have been demonstrated. Cell culture substrates made from fullerene assemblies are desirable for their well-defined structure and strong protein adsorption.

**Figure 8:**
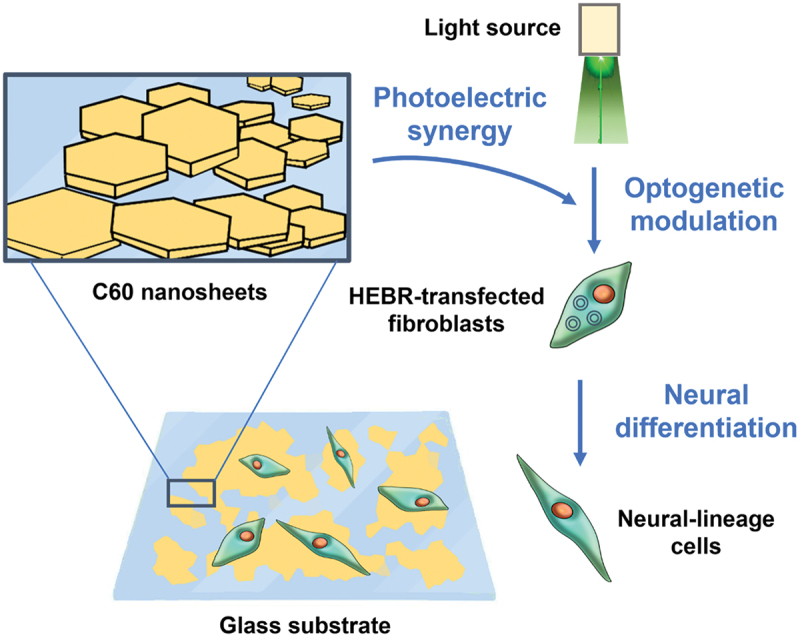
C_60_ nanosheets are used as a substrate for HEBR-transfected fibroblasts. the nanosheet substrate synergistically amplified optogenetic modulation of fibroblasts expressing the HEBR photon pump, resulting in increased differentiation to neural-lineage cells (as characterized by neural gene and marker expression). See text for details.

Fullerene nanosheets are two-dimensional crystalline assemblies of C_60_ fullerene shaped like thin hexagonal prisms. Fullerene nanosheets are prepared using LLIP at the interface between carbon tetrachloride (CCl_4_) and an alcohol. The size of nanosheets depends on the alcohol used; the hexagonal face of nanosheets produced with methanol, ethanol, and isopropyl alcohol (IPA) has diameters between 500–700 nm, 2–3 µm, and 7–9 µm, respectively, [188]. Luo et al. reprogrammed human fibroblasts to neural lineages through optogenetic modulation on a fullerene nanosheet platform [189] (Figure 8). Human fibroblasts were transfected with highly expressible bacteriorhodopsin (HEBR) genes, which produce photo-responsive proton pumps with the potential to trigger neural activity. C_60_ nanosheets prepared through LLIP (CCl_4_/benzene and IPA) were formed into Langmuir-Blodgett (LB) films on glass and polyurethane substrates. Transfected fibroblasts cultured on C_60_ nanosheet substrates and stimulated with light significantly upregulated neural lineage-related genes *GFAP*, *β-tubulin*, *MAP2* and corresponding marker proteins; transfected fibroblasts differentiated toward neural lineages without induction medium. Optogenetic modulation is synergistically enhanced by photoelectric properties of C_60_. Fullerene nanosheet substrates are promising for neural therapies based on optogenetics.

### 3.3. Two-dimensional assembly of one-dimensional fullerene nanowhiskers

Fullerene nanowhiskers (FNWs) are one-dimensional crystalline fibers assembled from C_60_ fullerene. FNWs are prepared using LLIP at the interface between toluene (or *m*-xylene) and an alcohol (usually IPA) [190–192]. FNWs used in cell culturing typically have cross-sectional diameter between 300 and 500 nm; FNW lengths range between 1 and 500 µm depending on fabrication parameters, representing a wide range of aspect ratios.

**Figure 9:**
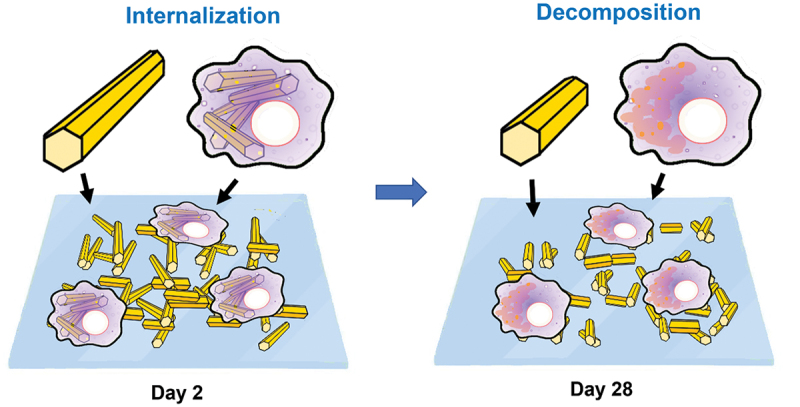
Macrophage-Like cells internalize FNWs within 2 days. Within 28 days, average fullerene nanowhisker length became shorter, and granular crystallizations appeared in cell membranes, indicating decomposition by macrophages. Cell interactions with fullerene nanowhiskers induced internalization and decomposition.

Nudejima et al. co-cultured macrophage-like cells with FNWs, observing phagocytosis and decomposition into fullerene molecules [193,194] (Figure 9). FNWs (toluene and IPA) were dispersed in culture medium (10 µg/mL). After 2 days of exposure, >70% of cells internalized FNWs. After 28 days, short FNWs became more numerous, and granular crystallizations appeared in cell membranes. This suggests that cell interactions with FNWs may involve internalization and decomposition, as opposed to simply adhesion. The result also demonstrates a potential for FNWs in drug delivery systems and tissue engineering. In a follow-up study, Okuda-Shimazaki et al. found very weak cytotoxicity associated with macrophage-like cells internalizing FNWs, lower than multiwall carbon nanotubes (MWCNTs) at the same concentration [195].

**Figure 10:**
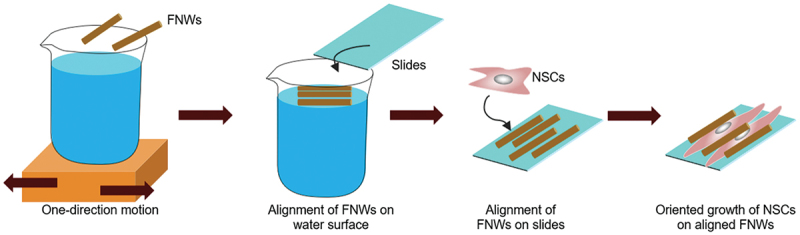
FNWs were aligned with one-direction motion by the lab shaker and captured on a glass slide to produce aligned FNWs scaffolds. When used as substrates for NSCs culture, NSCs tend to orient along the direction FNWs. Morphological changes of NSCs triggered neural differentiation.

Methods to organize one-dimensional FNWs into two-dimensional arrays of controlled geometry at air–water interfaces were also developed. The organized assemblies are easily transferred to solid substrates. As cell culture substrates, FNW arrays exert effective control over cell adhesion, morphology, and differentiation. Krishnan et al. used vortex-aligned FNWs to control the orientation of human osteoblast MG63 cells [196]. FNWs (toluene and IPA) were added to a water surface in a uniform layer. Mechanical stirring causes vortex flow that orients FNWs with the motion. FNWs are transferred to a substrate during motion. Length and packing density of FNWs, distance from the center of rotation (during transfer), rotation rate, and substrate hydrophobicity tune the geometry of FNWs arrays. MG63 cells preferentially adhered to FNWs, growing along the axes of alignment; this strongly influenced cell morphology. Cell adhesion favours FNWs due to enhanced adsorption of extracellular matrix (ECM) proteins and increased surface roughness. Cell proliferation on FNWs and glass was comparable, indicating low toxicity. These results demonstrate potential for two-dimensional assembled FNW substrates to promote adhesion and guide cell growth. In a similar approach, Hsieh et al. used cyclic linear motion to produce aligned FNWs (toluene and IPA; m-xylene and IPA), which promoted differentiation in murine NSCs (Figure 10) [197].

**Figure 11:**
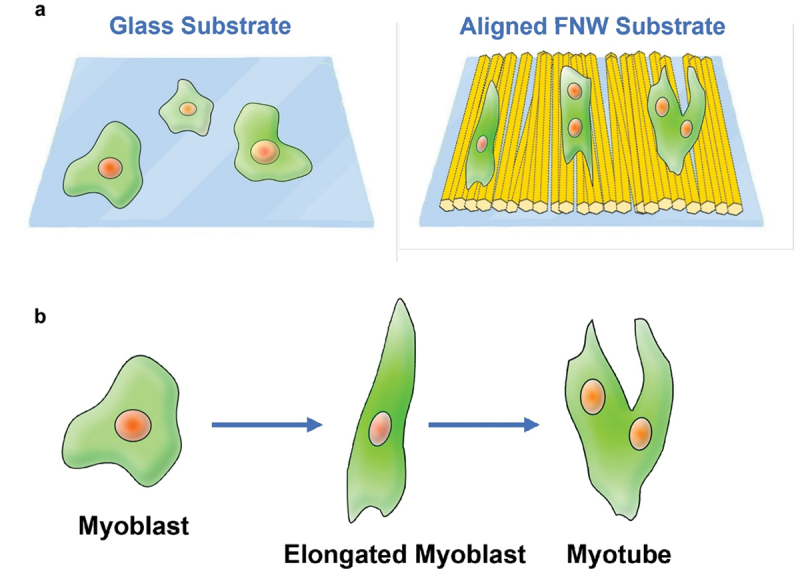
A) on aligned FNW substrates fabricated with the LB technique, myoblasts elongated and oriented along FNW alignment. Over time, some cells fused, becoming multinucleated, a characteristic representative of mature myotubes. b) Elongation induces an early stage of differentiation; fusion of elongated cells occurs at the late stages of differentiation.

Lateral compression in the LB technique spontaneously aligns FNWs parallel to barriers. Minami et al. used the LB approach to fabricate aligned FNWs, which stimulated myogenic differentiation in murine skeletal myoblasts [198] (Figure 11). FNWs (toluene and IPA) were compressed against and transferred to glass slides, forming LB films of aligned FNWs achieving high surface coverage of 79%; the films display consistent geometry independent of substrate properties (i.e., hydrophobicity). Myoblasts took on elongated morphologies (aspect ratio 1.78-fold that on glass), with growth direction and orientation highly correlated to FNW alignment. Myoblasts significantly upregulated myogenic genes *MyoD* and *Myogenin*. Myoblast fused to form multinucleated phenotypes characteristic of mature myotubes. The topography of aligned FNWs regulated cell morphology and growth direction, which induced myogenic differentiation.

**Figure 12:**
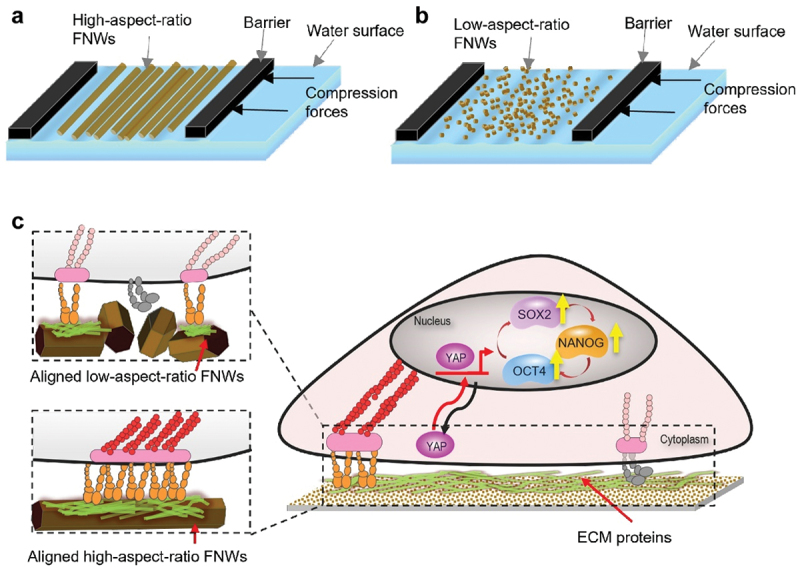
Tuneable aligned FNWs scaffolds regulate stemness genes expression of hMSCs. a) LB technique aligns high-aspect ratio FNWs parallel to barriers, forming aligned FNWs arrays. b) Alignment decreases with low-aspect ratio FNWs. Adjusting the ratio of different FNWs tunes alignment on a continuous range. c) Aligned FNWs provide appropriate cues for mechanotransductive upregulation of stemness genes through the YAP signalling pathway.

Song et al. effected tunable alignment of FNWs assembled through the LB approach. Improved control over nanoarchitecture enabled more detailed correlation between FNW alignment and cell behaviours. High alignment promoted self-renewal and retention of multipotency in human mesenchymal stem cells (hMSCs) (Figure 12) [199]. FNWs (m-xylene and IPA) of different aspect ratios were fabricated by controlling addition rate (of IPA to C_60_/m-xylene solution). Adjusting the composition of FNWs (high aspect ratio: low aspect ratio) tuned their alignment in the resulting LB film. With increasing alignment, hMSCs exhibited increasing unidirectional orientation (along FNWs) and elongation. Upregulation of stemness genes *OCT4*, *SOX2*, *NANOG* and corresponding markers correlated with increasing alignment. Hallmarks of stemness persisted over 2 weeks, indicating long-term multipotency retention through symmetrical self-renewal. This result may become significant for *in vitro* stem cell expansion, which is desirable in tissue engineering and clinical applications. The hydrophobic and nano-topographic surface of aligned FNWs provided appropriate cues to restrict focal adhesion growth and activate mechanotransducive Yes-Associated Protein (YAP) signalling pathways; YAP nuclear translocation promoted upregulation of stemness genes, modulating cell fate.

Cell substrates made from two-dimensional FNW assemblies modulate cell morphology and differentiation, as shown with osteo, neural, muscular, and stem cells in featured reports. Novel self-assembly processes progressively improved control over nano-topography, producing well-controlled cell experiments and meaningful results for tissue engineering applications.

## 4. New frontier of two-dimensional environment for bio-related applications: liquid-liquid interface

Artificial cell substrates are almost exclusively solids (glass, metals, and polymers). Still, cells growing and maintaining vital functions on liquid substrates has been demonstrated. Water-immiscibility allows a liquid to form a phase boundary with tissue culture medium. If the liquid is also denser than water, then it will form the bottom phase. Liquid–liquid interface culture (LIC) grows cells at the phase boundary. Cells are supported on the bottom phase (water-immiscible liquid) and receive nutrients from the top phase (medium). Liquid–liquid interface culture offers unique insights into cell-substrate interactions and unique conditions for modulating cell behavior. Elastic stress is nearly absent as compared to solid substrates. Viscous stress ranges widely depending on liquid properties. These factors create atypical cell environments, where cell behaviors can be strongly correlated to viscoelastic substrate properties.

### 4.1. Fluorocarbon–medium interface

Cell culture at the interface between perfluorocarbon (PFC) oil and culture medium was first described by Rosenberg in 1964 [200–202]. Perfluorocarbons are inert hydrophobic liquids; the immiscible interface between perfluorocarbon and medium can support anchorage-dependent cells. The interface also sustains cells, owing to perfluorocarbons’ high gas solubility and low toxicity. It is important to note that cells do not adhere directly to the interface, but instead to serum proteins (recruited from medium) or artificial microcarriers adsorbed at the interface [202–205]. Due to weak interactions, proteolytic enzymes or chelating agents are unnecessary to detach cells. Hence, passaging becomes simple and preserves intact membrane proteins [206]. This forms a defining advantage over solid substrates. Nonetheless, cells grown on perfluorocarbon-medium tend to aggregate and exhibit reduced growth and spreading [207]. Initially, the perfluorocarbon-medium liquid interface culture was done exclusively on fibroblasts because they facilitate their own attachment and growth by secreting large quantities of collagen and fibronectin [208]. Sanfillipo et al. enabled fibroblast and epithelial cell growth on perfluorocarbon with transforming growth factors (TGFs), but cell aggregation and poor spreading remained significant issues [209].

**Figure 13:**
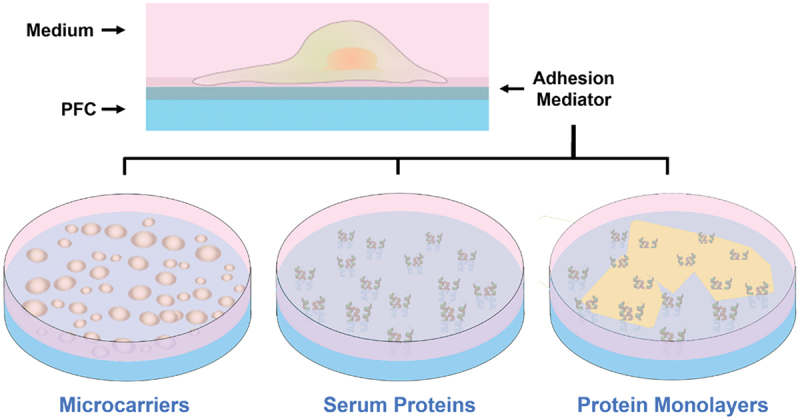
Ostensibly, liquid-liquid interface culture (LIC) involves cell adhesion and growth at the interface between a perfluorocarbon (PFC) and culture medium; but cells actually adhere to molecules adsorbed at the interface. Reported adhesion mediators include emulsion-based microcarriers, serum proteins recruited from medium, and pre-assembled protein monolayers.

In 1983, Giaever and Keese made a breakthrough in introducing cell culture on pre-assembled protein monolayers at the perfluorocarbon–medium interface (Figure 13) [210]. The monolayer self-assembles after simply depositing matrix protein solutions on perfluorocarbon and incubating. The monolayers are further enriched by adsorbing serum proteins from medium. Giaever and Keese cultured murine and human fibroblasts on poly-L-lysine (PLL) monolayer, successfully growing confluent cell sheets [210]. Ando et al. created collagen type IV (COL), laminin (LN), fibronectin (FN), fibrinogen (FG), and gelatin (GN) monolayers, observing endothelial cell growth comparable to that on polystyrene [211]. Kwon et al. noted significantly improved fibroblast growth and spreading on PLL and GN monolayers [212]. Improvements in spreading may be related to a protein monolayer’s tensile strength [210–212]; aggregation has been associated with monolayer fracturing caused by cell-exerted tension [202,210].

Perfluorocarbon composition leads to significant differences in cell spreading and morphology. FC70 [202,207,210], FC72 [202,210], FC40 [205,207,213], FC43 [202,208,209,211], and perfluorodecalin (PFD) [207,214,215] support palpable cell growth and spreading, while FC-77[202,204,210,212], L-series [202], and KPF-series [207] do not. High-purity perfluorocarbons tend to inhibit spreading, suggesting endogenous contaminants may act as surfactants modulating protein adsorption ability [207,209,210]. This has been further evidenced in studies using exogenous surfactants [202,216]. Perfluorocarbon structure may also influence cell morphology. Minami et al. tested a linear aliphatic perfluorocarbon (perfluorooctane (PFO)), a cyclic perfluorocarbon (undecafluoro-tryifluoromethylcyclohexane), an aromatic perfluorocarbon (hexafluorobenzene), and various mixtures of these perfluorocarbons, finding cell spreading only on neat PFO [217]. Some perfluorocarbons also exhibit cell-specific inhibitory effects or toxicity [201].

**Figure 14:**
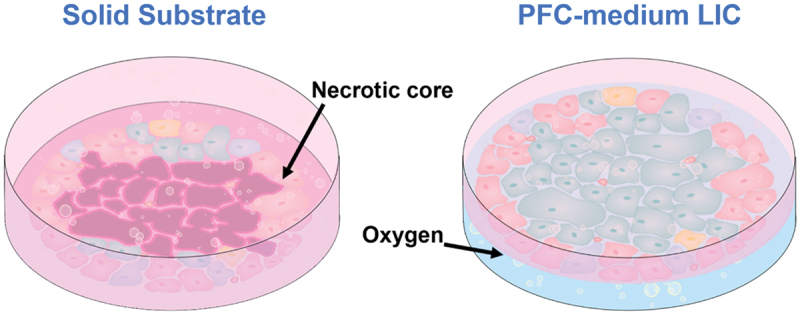
High-Density cell clusters such as islets are prone to hypoxia. Oxygen diffusion to the islet core is limited, particularly on solid substrates; central necrosis ensues. Perfluorocarbons (PFCs) have high gas solubility. PFC-medium liquid-liquid interface culture (LIC) offers pan-directional oxygen delivery, encouraging islet survival and improved function.

Perfluorocarbon-medium liquid interface culture has significant implications for tissue engineering because it enhances respiratory gas transport, which is an issue for high-density cell clusters. For example, large islets cultured on plastic substrates tend to suffer hypoxic damage due to reduced oxygen supply, leading to necrosis. Culture at the perfluorocarbon–medium interface offers unique advantages in pan-directional oxygen delivery (Figure 14). Juszczak et al. cultured islets on PFD, preserving murine islet viability for 72 h and improving post-transplant insulin secretion [214]. The 72 h viability of liquid–liquid interface culture islets reached 79% compared to 53.4% on plastic; islets on plastic contained large necrotic cores, which were absent in liquid interface culture islets. Liquid–liquid interface culture islets secreted significantly more insulin in response to glucose stimulation. This was also reflected post-transplantation, as 50% of liquid–liquid interface culture islet recipients became nonglycemic, compared to 14.3% of plastic-cultured islet recipients, and 31.3% of fresh islet recipients. Besides islets, liquid–liquid interface culture oxygen delivery is found to benefit chondrocyte clusters on polylactide scaffolds [218].

Substrate viscoelasticity strongly influences cell adhesion, morphology, and differentiation [219–223]. Effective absence of viscoelastic stress in perfluorocarbon-medium liquid interface culture has interesting implications. Minami et al. cultured C2C12 myoblasts at perfluorocarbon–medium interfaces, finding suppression of myogenic differentiation even in differentiation medium (DM) [216]. Myoblasts cultured on polystyrene upregulated myogenic genes *myoD*, *myf5*, *myogenin*, and muscle-specific gene *MHC*, indicating differentiation into myotubes. Myoblasts cultured on PFO-DM retained high viability and spread but only upregulated *myoD*; absent viscoelastic stress in liquid–liquid interface culture weakened cellular traction force (CTF), causing mechanotransducive *myf5* and *myogenin* downregulation, which suppressed myogenic differentiation.

The behavior of stem cells in liquid–liquid interface culture is of interest for tissue engineering and clinical applications. Hanga et al. cultured hMSCs using liquid–liquid interface culture, citing benefits of membrane protein preservation for stem cell expansion [213]. FC40 was pre-conditioned by incubating with growth medium. This was sufficient for good transient attachment; within 2 h, hMSCs assumed their characteristic spindle-like morphology, as they would on plastic substrates; hMSCs were well spread and highly viable (>90%). However, over 7 days, liquid–liquid interface culture cell numbers and growth kinetics were lower (>2.5 fold) than on polystyrene, indicating suppressed cell activity. Importantly, liquid–liquid interface culture hMSCs retained their stemness markers, pluripotency, and differentiation potential post-expansion.

**Figure 15:**
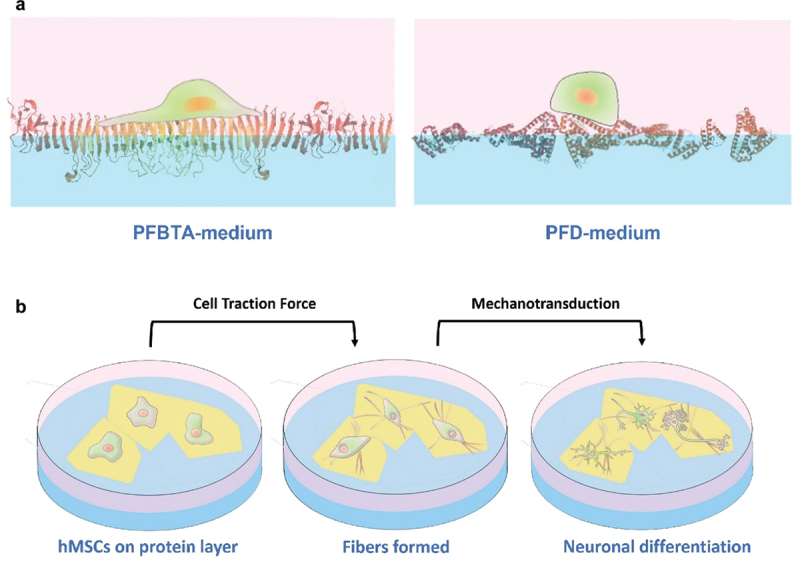
a) Stronger protein denaturation on PFBTA (compared to PFD) creates a densely packed protein monolayer; the monolayer resists CTF, so it is perceived by cells as a stiffer substrate. on PFBTA, hMSCsspread, formed strong focal adhesions, and activated mechanotransducive signalling. on PFD, hMSCs only formed dynamic adhesions. b) on PFO, CTF exerted by hMSCs deformed protein monolayers into one-dimensional fibers. the fibers influenced hMSC morphology, activating mechanotransducive neuronal differentiation.

Jia et al. discovered that the behavior of hMSCs on pre-assembled protein monolayers depends heavily on cell-perceived stiffness (Figure 15) [224]. Proteins assembled on different perfluorocarbons exhibit different packing densities; resistance to deformation by CTF is perceived by cells as stiffness. Stiff protein layers (128 kPa) formed on perfluorotributylamine (PFBTA) promoted cell spreading, strong focal adhesions, and YAP nuclear translocation (evidencing mechanotransduction). Soft protein layers (92 kPa) formed on PFD impaired focal adhesion growth and cell spreading. Previous studies reported fractured protein layers caused by cell-exerted tension, which in turn caused cell aggregation [202,210]. In Jia et al.’s follow-up work, hMSCs significantly deformed protein monolayers formed on PFO, triggering a feedback mechanism that altered cell morphology and fate (Figure 15) [225]. The protein layer (96 kPa) transformed into elongated one-dimensional fibers under CTF-induced interfacial jamming. The elongated fibers coincided with elongated focal adhesions, followed by hMSCs adopting neuronal morphology. Significant upregulation of neuronal genes *MAP2* and *TUBB3* and corresponding markers further evidence neuronal differentiation. This mutual modulation between cells and protein layers is reminiscent of feedback mechanisms in tissue morphogenesis. Biomimetic stem cell regulation using liquid–liquid interface culture may be an interesting pursuit for tissue engineering.

### 4.2. Viscous liquid interface

Formation of short bonds between polymer chains, known as crosslinking, is an effective way to modulate material viscoelasticity. Some weakly crosslinked or non-crosslinked polymers are effectively viscous liquids [216]. Cell culture at the interface between medium and such viscous liquids has been demonstrated. Kong et al. studied the rheology of weakly crosslinked and non-crosslinked poly(dimethyl siloxane) (PDMS), observing full stress relaxation (i.e. classical viscous liquid rheology). HaCaT epidermal cells grew and spread on non-crosslinked PDMS; adhesion appears to be mediated by adsorbed serum proteins, as is the case for perfluorocarbons [216]. Sylgard 184 PDMS supported cell adhesion, while neat PDMS with comparable viscosity did not. Additives in Sylgard 184 PDMS may have acted as surfactants mediating protein adsorption to the medium-PDMS interface.

**Figure 16:**
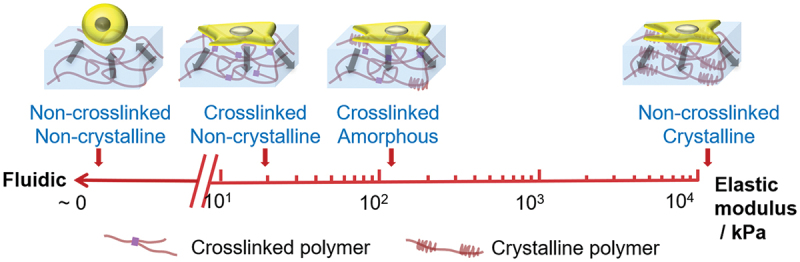
CL-DLLA copolymer has adjustable viscosity and elasticity. Non-crosslinked polymers exhibit viscous liquid rheology. Viscosity increases with crosslinking, and elasticity arises from crystalline regions. As a cell substrate, CL-DLLA copolymer’s adjustable viscosity and elasticity regulates cell spreading. Fibroblast spreading increases significantly with crosslinking, and more gradually with elasticity. This leads to the conclusion that cells may be significantly more sensitive to substrate viscosity than elasticity. See text for details.

Uto et al. created a copolymer of caprolactone (CL) and D,L-lactide (DLLA) with adjustable viscosity and elasticity; substrate viscosity played a bigger role in cell spreading than previously understood [223] (Figure 16). Elasticity of CL-DLLA copolymer was controlled by adjusting the CL/DLLA composition. Viscosity was controlled with crosslinking. Non-crosslinked CL-DLLA was immiscible with medium and exhibited viscous liquid rheology. The interface was primed with FN coating before seeding with NIH3T3 fibroblasts. Cells remained spherical on liquid-like CL-DLLA, becoming increasingly well spread as viscosity and elasticity increased. On liquid substrates, dissipation of CTF prevents formation of focal adhesions and actin stress fibers. Fibroblast spreading increased significantly with crosslinking, and more gradually with elasticity; cells may be significantly more sensitive to substrate viscosity than elasticity, contrary to established notions [219–222]. In the CL-DLLA substrate, relative viscous and elastic properties are easily adjusted. Cell responses to viscosity can be isolated and contrasted with responses to elasticity, uncovering new dimensions in biomaterials.

## 5. Short-term perspectives

In this review article, several examples of bio-interactive nanoarchitectonics with two-dimensional materials and two-dimensional environments (interfaces) are presented. Although these examples cannot cover all related aspects, general trends and insights can be found therein. Interfaces are important conduits for transmitting nanoarchitected material/structural information to bioactive systems including living cells. Extending beyond intrinsic properties of the interfacial environment, two-dimensional materials and two-dimensional material assemblies effectively modulate bio-interactive capabilities at the interface. A distinct feature is information relay with incredible functional amplification and scaling. A small interactive cue created at the interface can propagate to trigger vital functions such as cell differentiation. Simple material interactions can decide the fates of living creatures. This excellent information processing is built upon sophisticated organization of functional molecules and their rational signalling pathways. In many ways, it is a brilliant demonstration of the ultimate goal of nanoarchitectonics approaches for functional materials systems. Production of highly advanced functional materials based on bio-like sophisticated component organization is a quintessential task for materials nanoarchitectonics [226], a task enabled by architecting nanoscale components as the post-nanotechnology methodology. Unlike naturally occurring systems, nanoarchitectonics processes benefit from much wider component selections from non-bio materials such as two-dimensional materials. Therefore, there is even greater potential to create functional materials. Emerging fields such as materials informatics [227–229] could support the development of functional materials systems from such a wide pool of components and selection freedoms.

While nanomaterials possess much potential for interesting applications through nanoarchitectonics, consumer and work-related exposure to nanomaterials have some safety concerns. Some of these common nanomaterials like silica, titanium dioxide and zinc oxide nanoparticles are added to consumer products like powdered food products and sunscreens. These nanomaterials have been shown to elicit pro-cytotoxic, inflammatory, pro-carcinogenic and dysfunctional outcomes in a variety of cell types present in various major organs [230–238]. It would then be critical that with our growing understanding of the negative effects of nanomaterials, we can then use nanoarchitectonics design principles and tools to avoid the negative imprinting of nanomaterialistic features into next-generation nanomaterials to build functional bionanomaterials that are safe for the humans and the environment.

Nanoarchitectonics has mostly focused on engineering new materials up to this date, and it is on an exponential trajectory of growth. Much of this growth is based on wet laboratory experimentation studying interactions between molecules. Experimental parameters accumulated over decades of nanoarchitectural synthesis research can be fed as machine learning datasets. Machine learning algorithms can data-mine for patterns to propose combinations of parameters that may have been overlooked by real bench synthesis studies. These in-silico methods will accelerate the development cycles for new materials from design to actual proofs-of-concept. As a general outlook, metaphysical tools together with bench-based synthesis methods may propel nanoarchitectonics to the next stage of growth as we strive to search, design, apply and produce new nanomaterials for societally beneficial applications.
